# Non-compacted, PET-insensitive amyloid states increase after systemic inflammation and predict neuritic damage across Aβ pathology models and Alzheimer patients

**DOI:** 10.21203/rs.3.rs-8269406/v1

**Published:** 2026-01-13

**Authors:** Ping Liu, Ann-Christin Wendeln, Jessica Wagner, Fabian Brückner, Jian Sun, Xiaoqin Huang, Thomas S. Lewis, Lisa Steinbrecher, Nina Hermann, Xidi Yuan, Yuanyuan Deng, Angelos Skodras, Natalie Beschorner, Therése Klingstedt, Katleen Wild, Lisa Häsler, Marius Lambert, Simon Lindner, Tammaryn Lashley, Matthias Brendel, K. Peter R. Nilsson, Mathias Jucker, Jonas J. Neher

**Affiliations:** 1 German Center for Neurodegenerative Diseases (DZNE), Germany.; 2 Department of Cellular Neurology, Hertie Institute for Clinical Brain Research, University of Tübingen, Tübingen, Germany.; 3 Graduate School of Cellular and Molecular Neuroscience, University of Tübingen, Tübingen, Germany.; 4 Department of Neurology, The First Affiliated Hospital of Chongqing Medical University, Chongqing, China.; 5 Biomedical Center (BMC), Biochemistry, Faculty of Medicine, LMU Munich, Munich, Germany.; 6 Department of Nuclear Medicine, LMU University Hospital, Munich, Germany.; 7 NIAID Collaborative Bioinformatics Resource (NCBR), National Institute of Allergy and Infectious Diseases, National Institutes of Health, Bethesda, MD, USA.; 8 Division of Intramural Research (DIR), National Library of Medicine (NLM), National Institutes of Health (NIH), Bethesda, MD, USA.; 9 Department of Physics, Chemistry and Biology, Linköping University, Linköping, Sweden.; 10 UCL Queen Square Institute of Neurology London WC1N 3BG UK.; 11 Munich Cluster for Systems Neurology (SyNergy), Munich, Germany.

## Abstract

Neuroinflammation is a key modulator of Alzheimer’s disease (AD) risk, yet the impact of non-genetic inflammatory risk factors – such as systemic inflammation – remains poorly defined. Building on our previous work, here we show that 9 months after systemic lipopolysaccharide (LPS) challenge in APP23 mice, microglia-plaque interaction is disturbed and shifts Aβ aggregates toward a less compacted state, as revealed by conformation-sensitive amyloid dyes. Importantly, these structural changes are associated with increased plaque-associated neuritic dystrophy, phenocopying the effects of microglial risk genes. Generalising these findings, we show that across aging in APP23 and APPPS1 mice, and in AD patient tissue, non-compacted amyloid and microgliosis – but not compacted amyloid – are consistent predictors of neuritic damage. Notably, both in mouse and human tissue, *ex vivo* amyloid-PET signal largely reflects compacted but not non-compacted amyloid load. Our findings suggest that genetic and environmental risk factors converge on shared mechanisms of impaired microglial-plaque interaction and amyloid restructuring, and that commonly used amyloid-PET measures insufficiently capture amyloid states that define the severity of neuritic damage, with important implications for clinical trials in AD.

## Introduction

Genetic studies have linked alterations in the innate immune system to the pathogenesis of Alzheimer’s disease (AD)^1–4^. For instance, loss-of-function mutations in the gene encoding Triggering receptor expressed on myeloid cells-2, TREM2, preclude appropriate microglial responses to amyloid-β (Aβ) aggregation in mouse models and AD patients, resulting in reduced microglial association with Aβ plaques^5–7^. Similarly, mice lacking ApoE, whose isoforms are the most important genetic determinant of late-onset AD, show reduced molecular responses in microglia as well as limited microglia-plaque interaction^8–10^. Notably, deletion of *Trem2* or *Apoe* or introduction of their risk variants consistently results in less compacted amyloid and more neuritic dystrophy around plaques in mouse models^5,6,8,10^, indicating that reduced plaque compaction may be a common downstream mechanism that increases AD risk through enhancing plaque-associated neuronal damage. Accordingly, neuritic damage is a histological correlate not only of cognitive decline^11,12^ but also of key fluid biomarkers of neuronal injury downstream of plaque pathology, namely total tau and phospho-tau181^13–16^, in AD patients.

The effects of genetic risk on microglial activation and AD onset/progression have been studied in detail^7,17–19^; by contrast, the mechanisms of non-genetic risk factors — such as systemic infections and inflammatory diseases — remain poorly understood^3,20–22^. We previously described a potential mechanism of how systemic inflammation may affect AD pathology by demonstrating that peripheral immune stimulation results in long-term modification of the microglial epigenetic profile and, in turn, alters the microglial immune response to subsequently developing Aβ pathology in APP23 mice. This long-term microglial reprogramming was sufficient to modulate Aβ plaque burden at early disease stages^23^.

In this study, we further explored whether peripheral immune stimulation before the onset of Aβ pathology – and the resulting change in microglial responses – influences the structure and neurotoxicity of amyloid plaques in APP23 mice. To this end, we used conformation-sensitive amyloid-binding dyes — two luminescent conjugated oligothiophenes (LCOs), qFTAA and hFTAA – that bind β-sheet structures of distinct amyloid morphotypes^24^. These dyes allow analyses beyond the classical neuropathological assignment of *diffuse* vs. *neuritic/cored* plaques, which are robust for diagnostic scoring but provide only a binary readout and limited insight into fibril packing or plaque core vs. corona heterogeneity. In particular, qFTAA binds to tightly packed bundled Aβ-fibrils, i.e. highly compacted amyloid, while hFTAA binds to single filamentous as well as bundled Aβ-fibrils, but does not detect small non-fibrillar Aβ aggregates^24–26^. A combination of qFTAA and hFTAA dyes was previously used to demonstrate that plaques mature, i.e. become more compacted in APP transgenic mice with aging^27–29^ and that the lack of *Apoe* in APP transgenic animals leads to reduced plaque compaction^8^. Moreover, using these LCOs to analyse human tissue, Aβ plaques were found to be polymorphic in tissue from familial as well as sporadic AD patients^30^. Notably, a higher proportion of post-mortem hFTAA vs. qFTAA binding was also associated with more rapid progression of cognitive decline in AD patients^31^, indicating that hFTAA may detect more toxic amyloid species, but the mechanisms were not investigated.

Using qFTAA and hFTAA dyes, we here demonstrate that 9 months after systemic inflammation, microglial plaque interaction is altered, resulting in substantial restructuring of amyloid from compacted (qFTAA+) to non-compacted/filamentous (hFTAA+) aggregates in female APP23 mice. This, in turn, increased plaque-associated neuritic dystrophy — despite equivalent overall Aβ burden. We then extended our analyses across the life-span, both sexes, and an additional mouse model, as well as to tissue from familial and sporadic AD patients and confirm that increased non-compacted/filamentous amyloid is the main predictor of neuritic damage, whereas compacted amyloid appears largely inert or even protective across these conditions. Finally, using autoradiography with an FDA approved amyloid-PET ligand, [^18^F]flutemetamol, we find that this standard clinical measure of amyloid is largely reflective of compacted amyloid, failing to capture its more damaging filamentous forms.

Altogether, our findings indicate that genetic and non-genetic risk factors converge on shared disease mechanisms, namely the limitation of beneficial microglia-plaque engagement and a resulting shift of amyloid plaques to more neurotoxic forms. Moreover, our data show that diagnostics based on current amyloid-PET ligands alone may insufficiently capture amyloid morphotypes that define the severity of plaque-driven neuritic damage.

## Results

### Peripheral immune stimulation alters plaque morphotype in APP23 animals

In our previous work, we used peripheral stimulation with low-dose bacterial lipopolysaccharides (LPS) to model an acute vs. chronic peripheral inflammatory insult before the onset of Aβ pathology in female APP23 animals. We found that this triggered distinct and long-lasting microglial reprogramming as well as modulation of plaque burden at early pathology stages, i.e. in 9-month-old animals^23^. In particular, intraperitoneal (i.p.) injection of either a single dose of LPS (1xLPS; 500 *μ*g/kg bodyweight) or repeated injections of the same dose on 4 consecutive days (4xLPS) induced differential modulation of microglial immune responses to Aβ pathology, with 1xLPS increasing but 4xLPS decreasing pro-inflammatory microglial responses in 9-month-old APP23 animals. These findings reflected two previously described immune memory states, immune training and tolerance, in peripheral macrophages^32,33^.

**Figure 1:**
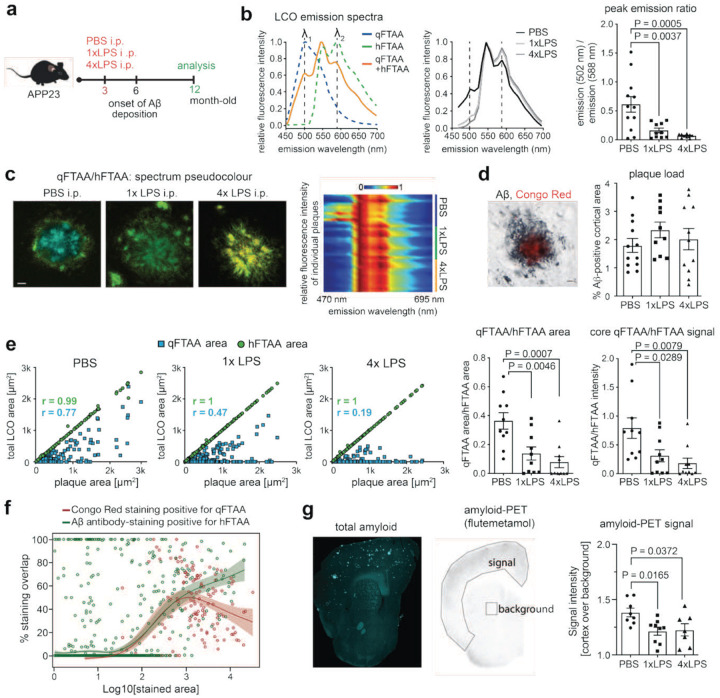
Systemic inflammation alters amyloid morphotype and PET-ligand affinity in APP23 mice. (a) Experimental timeline: APP23 mice were treated intraperitoneally (i.p.) with a single injection of bacterial lipopolysaccharides (1xLPS, 500 μg/kg), the same LPS dose on four consecutive days (4xLPS) or vehicle (PBS) at 3 months of age. Tissue was collected and analysed at 12 months. (b) *Left*: Principle of hyperspectral imaging with qFTAA and hFTAA staining: The emission ratio of 502 nm vs. 588 nm reflects the relative affinity of qFTAA (compacted amyloid) vs. hFTAA (filamentous amyloid). *Middle*: Emission spectra averaged across plaques show a shift in LCO fluorescence with 1xLPS and 4xLPS treatment. *Right*: Ratio of emission intensities at 502/588 nm of the average spectrum per animal (n=12,10,11 animals for PBS,1xLPS,4xLPS groups). (c) *Left*: Representative images of plaques from PBS/1xLPS/4xLPS-treated animals, pseudo-coloured based on LCO spectra. *Right*: Heatmap of collapsed emission spectra (cf. panel b) for individual plaques, indicating plaque heterogeneity in control (PBS) animals, and more homogeneous spectra after LPS treatment. (d) Immunohistological staining and quantification of plaques based on Aβ antibody staining, with Congo Red counterstain. The Aβ-positive cortical area is indistinguishable across groups (n=12,10,11 animals). (e) Correlation between total LCO-stained plaque area and individual qFTAA and hFTAA areas shows reduced correlation of qFTAA with plaque area in LPS-treated mice, suggesting less compacted plaques. Quantification of the average qFTAA/hFTAA area ratio and plaque core fluorescence intensity ratios per animal (n=12,10,11 animals for PBS,1xLPS,4xLPS groups) confirms hyperspectral plaque morphotype analysis. (f) Quantification of signal overlap between Congo Red (CR) and qFTAA (red curve) and between Aβ antibody (6E10) staining and hFTAA (green curve) highlights distinct binding properties of LCOs vs. classical amyloid staining approaches (3,115 plaques from n=5 animals). (g) Autoradiography of brain sections shows decreased affinity of the amyloid PET-ligand [^18^F]flutemetamol in 1xLPS- and 4xLPS-treated animals, reflecting decreased compact amyloid (values are mean for 3 sections each from n=8,9,7 animals for PBS/1xLPS/4xLPS groups). P-values are for posthoc Tukey test following significant one-way ANOVA. Data are presented as means ± SEMs.

As 9-month-old APP23 animals show early plaque pathology with many non-compacted amyloid aggregates, we here focused on 12-month-old animals to examine the impact of microglial immune memory states on plaque maturation and morphotype (Fig. 1a). We first used LCO hyperspectral microscopy to determine fluorescent signal intensities in the plaque core for the emission spectra peaks of qFTAA (502 nm) vs. hFTAA (588 nm) (Figs. 1b/c), as the commonly used measure of plaque morphotype^30^. Indeed, LCO hyperspectral imaging revealed significant alterations of Aβ plaque structure after 1xLPS and 4xLPS, with both treatments shifting amyloid aggregates to non-compacted/filamentous forms (as indicated by a decreased 502/588 nm emission ratio). Notably, while vehicle-injected APP23 mice contained a heterogenous mixture of plaque structures, both 1xLPS- and 4xLPS-treated animals had plaques with consistently reduced qFTAA affinity (Figs. 1b–c), indicating that peripheral immune stimulation triggers a shift in amyloid morphotype from naturally occurring heterogeneity to predominance of a non-compacted/filamentous amyloid structure.

**Extended Data Figure 1.**
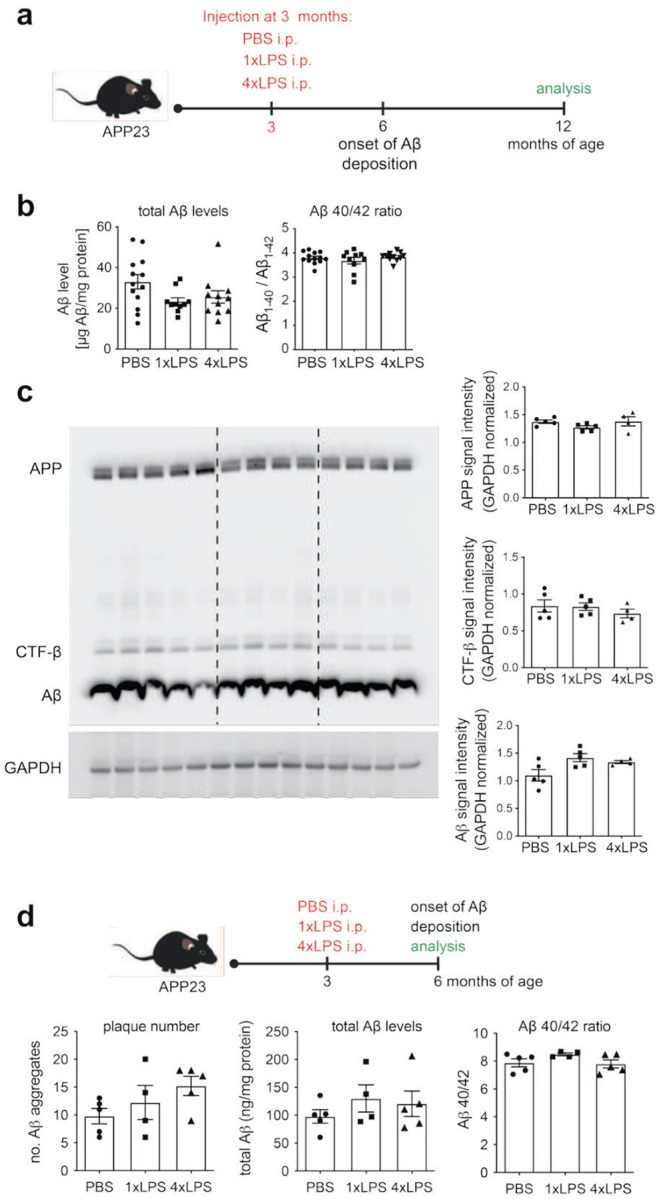
Systemic inflammation does not alter overall Aβ production or onset of plaque deposition in APP23 mice. (a) Schematic of the experimental design. (b) Quantification of total cortical Aβ levels and Aβ40/42 ratio by ELISA (n=11,9,10) shows no significant differences between treatment groups. (c) Representative Western blot and quantification of full-length APP, C-terminal fragment-β (CTF-β), and Aβ in brain lysates, normalised to GAPDH (n= 5, 5, 4 animals; values are the average of 4 independent Western Blots). LPS treatment does not affect levels of APP or its cleavage products. (d) Analysis of Aβ pathology at 6 months of age, following treatment with 1xLPS or 4xLPS at 3 months. Histological quantification of plaque number, and ELISA measurements of total Aβ levels and Aβ40/42 ratio (n=5,4,5) reveals no significant differences amongst treatment groups, indicating that peripheral immune stimulation does not affect pathology onset.

Alterations in the structure of amyloid plaques could occur as a result of differences in Aβ burden and/or plaque maturation. We have previously shown that 1xLPS and 4xLPS treatment led to an increase (for 1xLPS) or decrease in plaque load (for 4xLPS) by ~30% in 9-month-old APP23 animals. However, at 12 months of age, histologically determined plaque load (percentage of Aβ-positive cortical area; Fig. 1d), as well as biochemically determined total brain Aβ levels (Extended Data Figure 1b) were indistinguishable in 1x/4xLPS-treated mice vs. PBS-treated control animals. Furthermore, we could not detect changes amongst treatment groups in the ratio of brain Aβ_1–42_ to brain Aβ_1–40_ (Extended Data Figure 1b), excluding that different plaque structures occur as a result of altered proteolytic generation of Aβ isoforms. In addition, levels of amyloid precursor protein (APP) and C-terminal fragment (CTF-β) were equal between treatment groups (Extended Data Fig. 1c), indicating that Aβ processing itself was not changed. We also examined whether the onset of plaque deposition or the levels of Aβ differed amongst treatment groups in 6-month-old animals but could not detect any differences (Extended Data Fig. 1d), indicating that altered plaque morphotypes were not caused due to a shift in pathology onset, which might have affected plaque maturation.

**Extended Data Figure 2:**
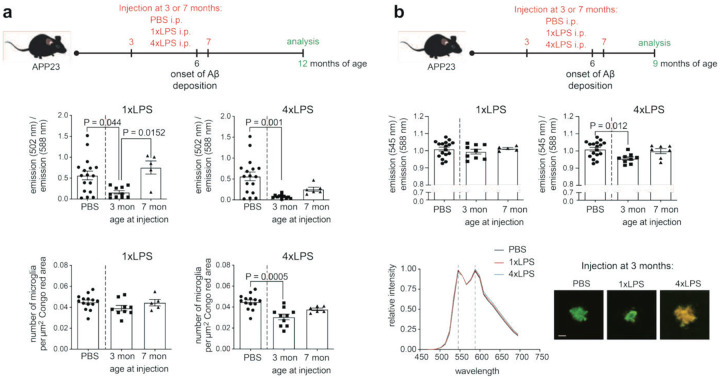
Length of time after systemic inflammatory insults determines its impact on amyloid morphotype and plaque-associated microglial numbers. (a) Experimental design: APP23 mice were intraperitoneally injected either at 3 months or 7 months with PBS (n=17, pooled for 3- and 7-months groups), 1xLPS (n=9/5 for 3/7 months injection), or 4xLPS (n=9/6 for 3/7 months injection) and analysed at 12 months. Quantification of LCO emission ratio (502/588 nm), and qFTAA/hFTAA area ratio shows that LPS administration at 3 months, but not at 7 months, significantly alters plaque morphotype. Stereological quantification of plaque-associated microglia for PBS (n=14), 1xLPS (n=9,5), and 4xLPS (n=10,6) treated animals stimulated at 3 or 7 months of age, indicates reduced microglial coverage only in mice that received 4xLPS treatment at 3 months. (b) APP23 mice were treated as in (a) but analysed at 9 months of age (n=17 for PBS, n=9,5 for 1xLPS at 3/7 months, n=9,7 for 4xLPS at 3/7 months), when immature plaques show virtually no binding for qFTAA and spectral analysis is based on the emission spectrum of hFTAA only. Only treatment at 3 months with 4xLPS alters plaque morphotype, as shown by decreased 545/588 nm ratios. Bottom row: Average emission spectra confirm a shift in fluorescence profiles in 3-month-old animals treated with 4xLPS. Representative images of LCO-stained plaques with spectral pseudo-colouring. Data are presented as mean ± SEM. P values are for Dunn’s multiple comparison following significant Kruskal-Wallis test.

We next asked whether the observed changes in plaque morphotype were due to an early change upon plaque deposition or due to slow restructuring during Aβ aggregation. To test this, we first compared 12-month-old animals treated with 1x or 4xLPS at 3 vs. 7 months of age. In animals injected at 7 months, plaques showed no (1xLPS) or notably smaller (4xLPS) effects on LCO spectra (Extended Data Fig. 2a). Similarly, when we analysed 9-month-old animals to capture early amyloid restructuring, changes in LCO spectra were detectable only in 4xLPS animals injected at 3 but not 7 months, and were undetectable in 1xLPS treated mice (Extended Data Fig. 2b). These results indicate that changes in plaque morphotype do not occur as an immediate result of LPS treatment/peripheral inflammation nor due to an early change in amyloid aggregation but rather require plaque restructuring, presumably through long-lasting alterations in microglial function.

While performing hyperspectral imaging, which requires spectral scans for each individual point of interest in the plaque (here performed in the plaque core), we noticed obvious changes also in the area covered by qFTAA vs. hFTAA staining. We therefore examined whether analysis of the area of the two LCO dyes would also reflect restructuring of plaques equivalent to hyperspectral analysis. In addition, we also compared shifts in the mean fluorescence intensity ratios of qFTAA/hFTAA in the plaque core. Indeed, when we quantified the LCO area ratio (qFTAA area/hFTAA area) or their plaque core intensity ratios, we obtained similar results, i.e. both 1xLPS and 4xLPS treatments at 3 months of age demonstrated shifts of both the area and plaque core intensity ratios towards higher hFTAA affinity in 12-month-old APP23 animals (Fig. 1e).

Since the qFTAA/hFTAA area ratio had confirmed the change in plaque morphotype at the scale of individual plaques (rather than single points of spectral measurements), we also examined in more detail how classical neuropathological stains, namely Congo Red and Aβ antibody staining relate to LCO affinity (using only PBS-treated 12-month-old APP23 animals to avoid confounds of experimental inflammatory changes). While qFTAA signal was found exclusively in plaque cores, it only partially overlapped with Congo Red staining. In particular, Congo Red stained smaller aggregates that were not recognised by qFTAA, and the signal overlap reached a maximum of ~50% for aggregates of intermediate size, but decreased again for large plaques (Fig. 1f). Similarly, hFTAA only partially overlapped with Aβ antibody staining, with hFTAA showing negligible affinity for very small Aβ deposits, but reaching up to 60–70% overlap with increasing aggregate size (Fig. 1f). In line with previous work^24–26^, these results indicate that qFTAA is binding only the most compact plaque components, while hFTAA selective recognises a non-compacted/filamentous amyloid state.

Next, we asked how plaque restructuring would affect the affinity of a clinically used amyloid PET-tracer. To this end, we chose [^18^F]flutemetamol, which — in contrast to other tracers such as [^18^F]florbetaben — binds measurably to diffuse, antibody-labelled Aβ aggregates; despite its higher affinity for compacted material, these diffuse aggregates can contribute substantially to net signal of the tracer^34–36^. Nevertheless, in APP23 brains, *ex vivo* [^18^F]flutemetamol autoradiography showed a significantly lower cortex-to-background signal in 1x and 4xLPS-treated animals (Fig. 1g), indicating that the tracer fails to detect the LCO-defined shift toward non-compacted/filamentous amyloid at equivalent total plaque burden.

### Plaque morphotype correlates with brain inflammatory state and microglial responses

**Extended Data Figure 3:**
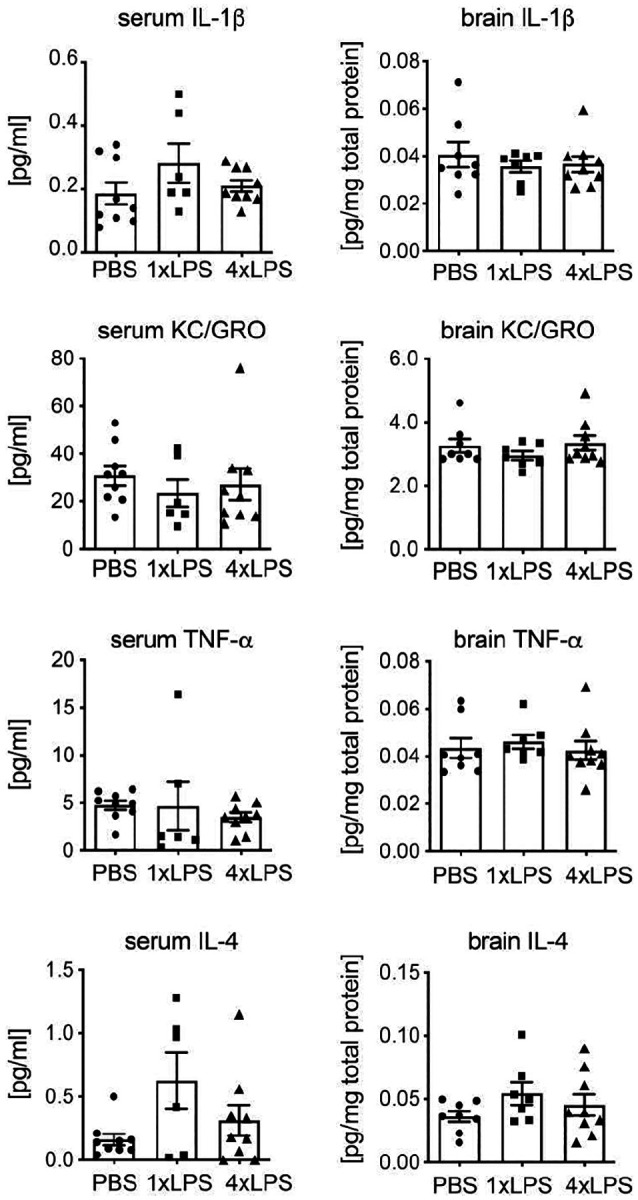
Levels of additional cytokines in 12 months old APP23 animals (related to Fig. 2). ELISA measurement of additional cytokines in serum (n=9,6,9 from PBS,1xLPS,4xLPS groups) and brain (n=8,7,9 from PBS, 1xLPS, 4xLPS groups) revealed no significant alterations between treatment groups. Data are presented as mean ± SEM.

**Figure 2.**
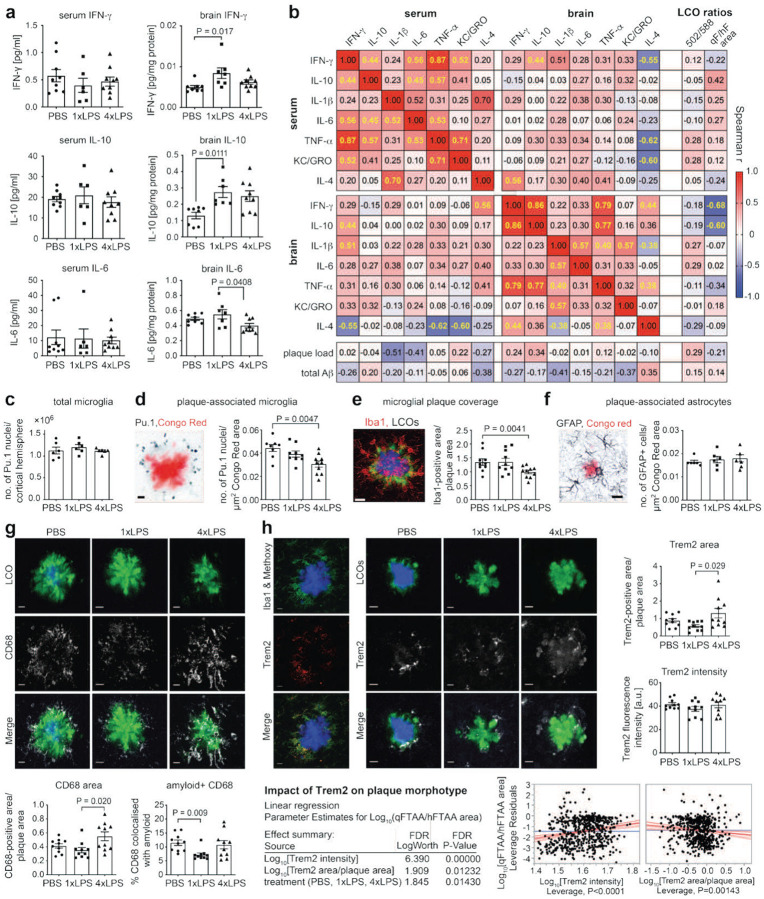
Systemic inflammation modulates cytokine levels, microglial plaque association and Trem2 engagement in APP23 mice. (a) Inflammatory cytokine levels in serum and brain. IFN-γ and IL-10 are elevated in the brains of 1xLPS-treated mice while IL-6 is decreased in the brain of 4xLPS-treated mice; levels of these cytokines in the serum are indistinguishable amongst treatment groups (serum n=9,6,9 and brain n=8,7,9 mice from PBS, 1xLPS, 4xLPS groups). (b) Correlations of cytokine levels and plaque load/Aβ levels with LCO-based plaque features indicate that increased levels of IFN-γ and IL-10 are associated with the shift to more filamentous amyloid, based on the qFTAA/hFTAA area ratio (significant correlations are indicated by yellow font). (c) Quantification of total cortical microglia (Pu.1+ nuclei) reveals no significant differences among treatment groups (n=6,6,6 animals for PBS, 1xLPS, 4xLPS groups). (d) *Left*: Representative image of plaque-associated microglia stained for Pu.1 and Congo Red. *Right*: Decreased density of plaque-associated microglia (Pu.1+ nuclei/μm^2^ Congo Red area) is observed in 4xLPS-treated mice compared to PBS controls (n= 8,9,10 animals for PBS,1xLPS,4xLPS groups). (e) *Left*: Representative image of Iba1+ microglia surrounding LCO-labelled plaques. *Right*: Iba1-positive coverage is reduced in 4xLPS-treated mice (n=10,10,10 animals for PBS, 1xLPS, 4xLPS groups). (f) *Left*: Representative image showing GFAP+ astrocytes around Aβ plaques. *Right*: Plaque-associated astrocyte density is indistinguishable across treatment groups (n= 6,6,6 animals for PBS,1xLPS,4xLPS groups). (g) *Top*: Representative images of CD68 staining colocalised with LCO-stained plaques. *Bottom*: CD68+ area is increased in 4xLPS-treated mice while co-localisation of CD68 with amyloid-staining is reduced in 1xLPS-treated animals (n=10,10,10 animals for PBS, 1xLPS, 4xLPS groups). (h) *Top*: Representative images of Iba1, Methoxy-X04 (amyloid), and TREM2 staining. *Right*: Trem2+ area per plaque is increased in 4xLPS-treated animals, while mean Trem2 fluorescence intensity is unchanged. *Bottom*, Linear regression for the effect of Trem2 area and intensity on plaque morphotype (qFTAA/hFTAA area ratio for n=691 plaques). Scatter plots show fitted regression lines and confidence intervals. Mean Trem2 intensity is positively associated with amyloid compaction, while increased Trem2 area shows a negative association. Scale bar = 10 μm. P-values are for Dunn’s posthoc analysis after significant Kruskal-Wallis test. Data are presented as means ± SEMs.

Since we could not find evidence for alterations in total Aβ plaque burden nor total Aβ levels, and because we have previously shown that microglial responses are differentially affected after 1xLPS and 4xLPS treatment^23^, we next examined whether alterations in plaque morphotype are related to a persistent modulation of cytokine levels in the brain and blood of 12-month-old APP23 animals. Using multiplex ELISA measurements for IFN-γ, IL-10, IL-1β, IL-6, TNF-*α*, KC/Gro and IL-4, we could not detect significant alterations in serum cytokine levels amongst treatment groups. In contrast, 1xLPS-treated animals exhibited increased brain levels of IFN-γ and IL-10 compared to PBS-treated controls, while 4xLPS-treated animals had reduced levels of IL-6 compared to 1xLPS-treated animals (Fig. 2a and Extended Data Fig. 3). We then asked whether the levels of particular cytokines in serum or brain correlate with alterations of plaque morphotype at the level of individual animals and across treatment groups. Interestingly, we found significant negative correlations between the qFTAA/hFTAA ratio and brain IFN-γ and IL-10 levels (which themselves showed a strong correlation), while other brain and serum cytokines showed no significant association with LCO parameters (Fig. 2b). Notably, neither cytokine levels nor LCO parameters correlated significantly with total plaque load (as measured by Aβ staining) or total Aβ levels (as measured by ELISA) (Fig. 2b), confirming that the change in amyloid structure was due to inflammatory mechanisms rather than total Aβ burden. These data indicate that peripheral immune stimulation modulates the brain inflammatory state for up to 9 months, with significant impact on amyloid compaction.

A number of studies have demonstrated that amyloid compaction depends on the so-called microglial barrier function, i.e. the microglial recruitment to and encapsulation of plaques^5,6,37^. Therefore, we next analysed microglial numbers and activation markers. Stereological quantification of microglia (based on nuclear Pu.1 staining) revealed no change in the total number of microglia amongst treatment groups (Fig. 2c); however, a significant reduction in the number of plaque-associated microglia was evident in 4xLPS treated animals (Fig. 2d), indicating reduced cell recruitment to plaques. Accordingly, analysis of whole cell staining demonstrated a decreased Iba1-positive microglial area after 4xLPS treatment (Fig. 2e). A less pronounced reduction of plaque-associated microglia was also apparent when animals were injected at 7 months of age, reflecting the magnitude of shifts in plaque morphotype (Extended Data Fig. 2a). In contrast, no alterations in the number of plaque-associated astrocytes were detectable (Fig. 2f).

As we had previously observed increased microglial Aβ phagocytosis in 4xLPS treated mice^23^, we next analysed lysosomal content in microglia (based on CD68 levels). Indeed, quantification of CD68-staining revealed a significant increase of CD68-positive area around plaques in 4xLPS- vs. 1xLPS-treated animals (Fig. 2g), despite the reduction in microglial number. Moreover, when we assessed co-localization of CD68 with LCO staining, we found a significant reduction in 1xLPS- but not 4xLPS-treated animals compared to controls.

The microglial barrier function is compromised in *Trem2* knockout mice and AD patients carrying *TREM2* risk variants, which consistently show impaired microglial recruitment to plaques, resulting in reduced amyloid compaction^5,6,37^. Therefore, we next analysed microglial Trem2 staining. In line with previous studies, we found Trem2 to be specifically localised to microglial processes contacting amyloid plaques (Fig. 2h). However, 4xLPS-treated animals showed less conspicuous Trem2 polarisation, presenting with a larger and more diffuse Trem2-positive area in the absence of a change in the mean fluorescence signal, while 1xLPS animals showed no discernible effects (Fig. 2h). To relate these findings more specifically to amyloid morphotype, we performed linear regression analysis at the individual plaque level, factoring in treatment, Trem2 intensity and Trem2 area to predict the qFTAA/hFTAA area ratio. Parameter estimates indicated that Trem2 mean intensity had the strongest impact on plaque morphotype, leading to more compacted amyloid; in contrast, increased Trem2 area, which likely reflects a lack of appropriate microglial process polarisation at the plaque surface, predicted a less compacted amyloid structure, with additional, independent effects of treatment also being apparent (Fig. 2h).

These data confirm our previous findings that peripheral inflammatory insults have a long-lasting impact on microglial responses to Aβ pathology. In addition, we now demonstrate that at more advanced pathology stages, 1xLPS and 4xLPS treatments exert differential effects on plaque-associated microglial number, lysosomal activity and Trem2 engagement. In both cases, this disturbance of appropriate microglial responses to Aβ deposition leads to a shift towards non-compacted/filamentous amyloid.

### Non-compacted/filamentous amyloid and microgliosis predict neuritic dystrophy in mouse models and AD patients

**Figure 3.**
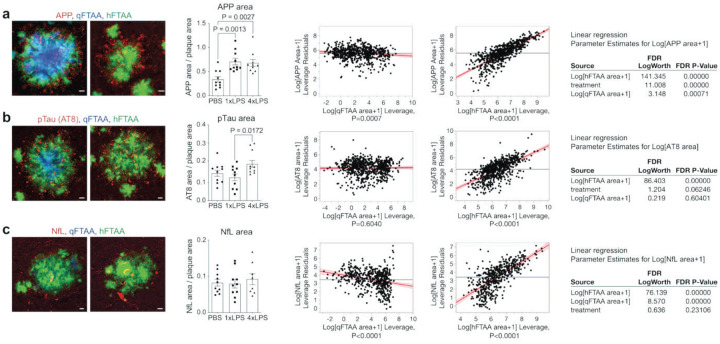
Filamentous but not compacted amyloid predicts neuritic dystrophy after systemic inflammation. (a-c), *Left:* Two representative images of plaques with higher (left) vs. lower (right) qFTAA/hFTAA ratio, co-stained with markers for neuritic damage. *Middle*: quantification of plaque-associated dystrophic neurites based on positive area of (a) amyloid precursor protein (APP, n=661 plaques), (b) phospho-Tau (pTau, n=686 plaques) or (c) neurofilament light chain (NfL, n=537 plaques) (n=10,10,10 mice for PBS,1xLPS,4xLPS groups). *Right*: Effect leverage plots and parameter estimates demonstrate the impact of qFTAA, hFTAA and treatment on markers of plaque-associated neuritic dystrophy in APP23 mice. Data are presented as means ± SEMs. P-values are for Dunn’s posthoc analysis after significant Kruskal-Wallis test. Scatter plots show fitted regression lines and confidence intervals. Scale bar 10 μm.

Since loss of plaque compaction is often associated with more neuronal damage in mouse models and patients carrying or lacking microglial risk genes, we next examined whether plaque-associated neuritic dystrophy was altered in LPS-treated animals. First, we assessed accumulation of amyloid precursor protein (APP) as a well-established marker of dystrophic neurites^38,39^. Indeed, both 1xLPS- and 4xLPS-treated animals showed exacerbated plaque-associated neuritic damage, as indicated by an increased area of APP-positive dystrophic boutons around plaques (Fig. 3a). In contrast, as a second marker of neuronal damage, phospho-Tau (pTau)^40^ was selectively increased in 4xLPS-treated animals (Fig. 3b), while neurofilament light chain (NfL), an alternative marker protein for neurodegenerative processes^41^, was unchanged amongst treatment groups (Fig. 3c). We then asked whether the two LCOs were predictors of neuritic damage at the level of individual plaques. Including data from all treatment groups (PBS, 1xLPS, 4xLPS), we found that for all markers (APP, pTau, NfL), hFTAA strongly predicted neuritic damage while qFTAA had no or even a negative impact, with additional treatment effects apparent for APP and pTau, as expected from our analysis at the animal level (Figs. 3a–c). This indicated that highly compacted, qFTAA+ amyloid is largely inert or even neuroprotective, while non-compacted/filamentous hFTAA+ amyloid is the primary mediator of amyloid-driven damage to nearby neurons. Notably, this relationship holds with modulation of plaque morphotype following peripheral inflammatory insults, establishing this association of non-compacted amyloid with increased neuritic damage beyond the impact of genetic factors (such as TREM2 and APOE)^5,6,8^.

**Figure 4.**
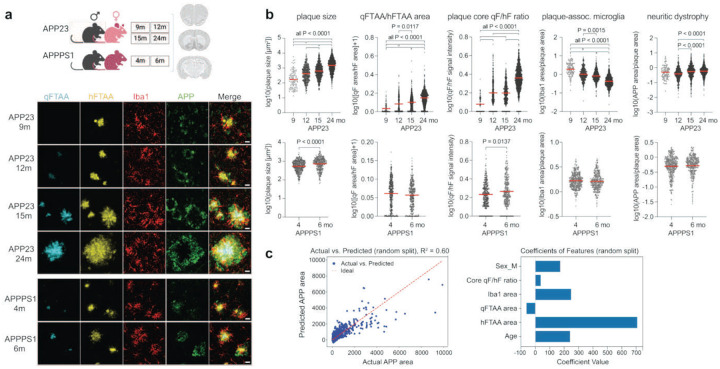
Filamentous but not compacted amyloid predicts neuritic dystrophy during aging and across mouse lines. (a) Schematic of experimental groups and representative images of qFTAA, hFTAA, microglia (Iba1), and neuritic dystrophy (APP) in APP23 mice aged 9, 12, 15, and 24 months, and APPPS1 mice at 4 and 6 months. (b) Quantification of plaque parameters in APP23 and APPPS1 mice (n=4–6 males and 4–6 females per age group). Age-related changes in plaque-associated microglial coverage (Iba1 area/plaque area) and qFTAA/hFTAA area ratio indicate progressive alterations in plaque structure and microglial-plaque interactions. The qFTAA/hFTAA area ratio increases with age, reflecting enhanced plaque compaction. Microglial-plaque association decreases while neuritic dystrophy increased with age in APP23 animals. (c) A multivariate regression model trained to predict plaque-associated APP area (neuritic dystrophy) showed good performance (R^2^=0.60). Feature importance analysis indicates that hFTAA-positive amyloid is the major predictor of neuritic dystrophy, while qFTAA-positive area even had a small negative effect. Data are presented as means ± SEMs; P values are for posthoc Tukey test following significant one-way ANOVA. n = 362, 309, 100, 391, 581, 1006 plaques for 4, 6, 9, 12, 15, 24-month groups, respectively. Scale bar = 20 μm.

Next, we examined whether the relationship between microglial-plaque association, plaque morphotype and neuritic damage is generalisable across mouse models, both sexes, and age groups in the absence of immune stimulation. We selected two mouse models, APP23 and APPPS1 mice, for comparison: These models generate different levels of Aβ peptides – with APPPS1 and APP23 mice generating Aβ42/Aβ40 ratios of 2.5–5.5 and 0.2–0.4, respectively^42^, resulting in different plaque morphotypes (Fig. 4a). In addition, plaque morphotypes have been shown to change with age; these conformational changes are detectable by LCO binding affinities^28^. Therefore, analysing 4–6 male and 4–6 female mice per group, we quantified qFTAA and hFTAA areas and their signal intensities in the plaque core, plaque-associated microglia, and dystrophic neurites in 4 and 6 months old APPPS1 mice (which develop first plaques around 6 weeks of age^43^) and 9, 12, 15 and 24 months old APP23 mice (which develop first plaques around 6–7 months of age^44^) for a total of ~2,750 plaques (Fig. 4b). As expected, in both models, plaque size increased with age, while microglial plaque coverage decreased relative to plaque size (Fig. 4b). In line with previous reports^28^, maturation of plaques was evident in APP23 mice by increased qFTAA staining, based either on the area ratio or the plaque core signal intensity ratio of qFTAA to hFTAA. In APPPS1 mice, plaque maturation from 4 to 6 months of age was also evident based on the plaque core signal intensity ratio of the dyes, but not detectable based on the area ratio. Notably, relative to plaque size, plaque-associated dystrophy increased with age in APP23 mice, while it remained stable in APPPS1 mice (Fig. 4b). We then applied support vector regression (SVR) – a supervised machine-learning method – to estimate the independent contributions of age, sex, LCO-based parameters and microglial plaque association to neuritic dystrophy. We trained and tested the model using a random split of 80:20% of the data and found that it could predict 60% of the variance in neuritic dystrophy (Fig. 4c). Notably, by far the strongest predictor of neuritic damage was the hFTAA area, whereas qFTAA area even had a negative impact, confirming the strong relative toxicity of non-compacted vs. compacted amyloid. Interestingly, plaque-associated microgliosis (Iba1 area) also showed a positive impact on neuritic dystrophy (Fig. 4c), suggesting that microglia contribute both to amyloid compaction but also to neuronal damage around plaques – in line with previous reports^45,46^.

**Figure 5.**
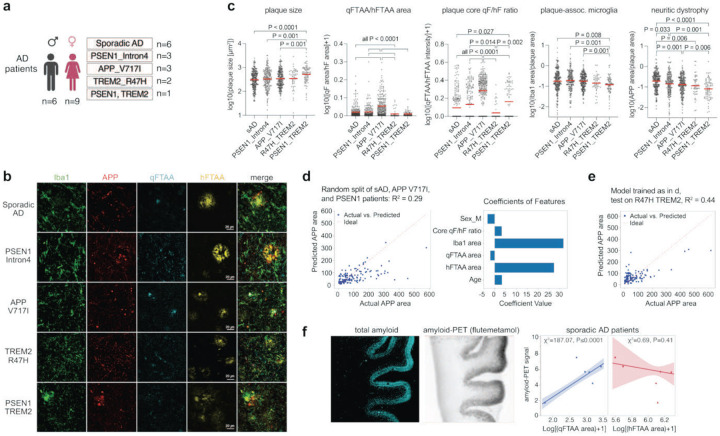
Filamentous but not compacted amyloid predicts neuritic dystrophy in sporadic and familial AD patients but is not captured by the amyloid PET-ligand [^18^F]flutemetamol. (a) Schematic overview of human brain samples analysed from individuals with sporadic (sAD) and familial AD (fAD), carrying *PSEN1 Intron4*, *APP V717I*, and *TREM2 R47H* variants as well as one patient carrying both the *PSEN1 Intron4* and *TREM2 R47H* mutations. (b) Representative confocal images show Aβ plaques stained with qFTAA (compact amyloid), hFTAA (filamentous amyloid), Iba1 (microglia), and APP (neuritic dystrophy). (c) Quantification of plaque metrics across patient groups. Plaque-associated microglial area (Iba1/plaque area) qFTAA/hFTAA area ratio is lower in *TREM2* mutation carriers, indicating a shift toward less compacted amyloid. APP-positive neuritic dystrophy per plaque is lower in all mutation carriers compared to sAD. (d) A multivariate regression model trained on sAD, *APP V717I*, and *PSEN1 Intron4* cases predicts plaque-associated APP area with reasonable accuracy (R^2^ = 0.29). Feature importance analysis reveals that both hFTAA area and Iba1 area are major contributors to model performance, and both positively predict neuritic dystrophy. (e) Testing the same model on the unseen cohort of *TREM2 R47H* patients (incl. the patient with PSEN1 mutation), improves predictive power (R^2^=0.44), in line with the known impact of *TREM2* mutations on microglial activation and amyloid compaction. (f) Autoradiography of brain sections using the amyloid PET-ligand [^18^F]flutemetamol tracks qFTAA+ but not hFTAA+ amyloid plaque components (robust Cauchy curve fit, with confidence intervals). Results are presented as means ± SEMs; P-values are for posthoc Tukey test following significant one-way ANOVA; n = 249, 185, 222, 52, 79 plaques for sAD, *PSEN1 Intron4*, *APP V717I*, *TREM2 R47H*, *PSEN1/TREM2*, respectively.

To examine the translational relevance of our findings, we analysed post-mortem tissue from the frontal cortex of 6 sporadic (sAD) and 6 familial AD patients, including 3 carriers each of the *APP V717* and *PSEN1 Intron4* mutation (n = 249, 222, and 185 plaques per patient group). Equivalent to our analysis in mice, we performed stainings for qFTAA, hFTAA, microglia (Iba1) and dystrophic neurites (APP) (Figs. 5a/b). While the average plaque size and microglial plaque-association did not differ amongst these patient groups, *APP V717* patients showed not only more compacted plaques than sAD and *PSEN1 Intron4* mutation carriers (based on qFTAA/hFTAA core intensity and area ratios) but also significantly lower neuritic dystrophy (Fig. 5c). To establish a general model of how plaque morphotype and microgliosis relates to neuritic damage in patients, we again trained an SVR model. Matching our data from mouse models, microgliosis (Iba1 area) and non-compacted/filamentous amyloid (hFTAA area) were the major predictors of neuritic damage, while compacted amyloid (qFTAA area) had a small negative/protective impact (Fig. 5d). However, in patients, the model was able to predict only 29% of neuritic dystrophy, likely due to patient heterogeneity and the relatively small sample size. Therefore, to validate our model, we additionally analysed tissue sections from 3 patients carrying the *TREM2 R47H* mutation (of which one patient carried an additional *PSEN1 Intron 4* mutation). The *TREM2 R47H* mutation is known to reduce microgliosis around plaques^6,40^, which we confirmed based on Iba1 staining (Fig. 5c). Therefore, model performance would be expected to improve in these patients due to biological modulation of this major predictor. Indeed, testing our model on data from the *TREM2 R47H* carriers improved prediction to 44% of variance in neuritic dystrophy in this subgroup of patients (Fig. 5e).

Finally, we tested the relationship between plaque morphotype and *ex vivo* PET-signal in the 6 sporadic AD patients. Across these samples, amyloid-PET signal covaried positively with qFTAA area but not with hFTAA area (Fig. 5f), consistent with our findings in mouse models. Thus, our results indicate that non-compacted, hFTAA+ amyloid predicts neuritic damage also in the human brain and that binding of the amyloid PET-ligand [^18^F]flutemetamol does not reflect this more damaging form of amyloid.

## Discussion

We here built on our previous work, where we demonstrated that systemic inflammatory insults can induce long-lasting epigenetic imprints in microglia, which modify their responses to later developing Aβ pathology^23^. In particular, we previously characterised the effects of a single vs. repeated LPS injections (1xLPS vs. 4xLPS) on early plaque deposition in 9-month-old APP23 animals. We found that 1xLPS reprograms microglia to respond with a heightened pro-inflammatory immune response to Aβ pathology, increasing plaque load. In contrast, 4xLPS induces long-lasting repression of inflammatory responses in microglia, resulting in increased microglial Aβ uptake and decreased plaque burden. Surprisingly, our present study revealed detrimental effects of both 1xLPS and 4xLPS at a later stage of pathology in 12-month-old APP23 mice. In particular, compared to controls, both 1xLPS- and 4xLPS-treated animals showed altered microglial plaque engagement and a substantial shift from compacted to non-compacted/filamentous amyloid, despite an indistinguishable total Aβ burden. Importantly, this change in plaque architecture was associated with exacerbated neuritic dystrophy (Figs. 1–3). Interestingly, similar effects have been described for animals with knockout of *Trem2*, which were found to have reduced Aβ pathology at early disease stages but exacerbated pathology at more advanced pathology states due to a suppression of microglial proliferation and their reduced association with plaques^47^. 4xLPS treatment phenocopied these differences in terms of reducing Aβ burden at early but not later stages of pathology, suppressing inflammatory responses to Aβ pathology, reducing proliferation and limiting plaque association of microglia (Fig. 2).

Extending these findings from immune-stimulated mice to naïve animals from two models (APP23 and APPPS1), both sexes, multiple ages, and tissue from sporadic and familial AD patients, we further show that non-compacted/filamentous amyloid (hFTAA+) is the strongest predictor of neuritic dystrophy, whereas compacted amyloid (qFTAA+) has no or even a protective effect. Since histologically-determined neuritic damage correlates with cognitive dysfunction^11,12^ as well as key fluid biomarkers in AD patients, namely total-tau and phosphor-tau181^13,14^, our work has several important implications:

First, our study highlights a failure of the microglial plaque barrier as a mechanism shared between genetic and non-genetic risk factors for late-onset AD, with long-term effects of systemic inflammatory insults resembling those of *TREM2* and *APOE* risk variants^5–9^. In particular, we find that systemic inflammatory stimuli trigger microglial immune memory states, which phenocopy genetic risk: acute (1xLPS) and repeated (4×LPS) immune stimulation both culminate in reduced plaque compaction, but by distinct mechanisms — higher inflammatory cytokines, altered plaque-associated phenotype (lower CD68) vs. fewer plaque-associated microglia and reduced Trem2 polarisation, respectively — both result in less compacted amyloid and worsened neuritic injury (Figs. 2/3). These observations dovetail with extensive work tying TREM2 and APOE to plaque compaction and neuritic dystrophy and may explain how infections increase the long-term risk for dementia^3,21^. Thus, our findings support therapeutic strategies that promote microglial compaction of plaques, e.g., through enhancing TREM2 signaling^48^.

Second, our data show that conformation-sensitive amyloid dyes can distinguish inert and neurotoxic forms of amyloid, which routine stains and PET ligands do not. Standard neuropathological methods define neuritic plaques morphologically, but they do not quantify compactness or conformational states. Our qFTAA/hFTAA approach transforms plaque typing into a quantitative spectrum, mapping plaque core vs. corona structure and allowing plaque-level modeling of neuritic injury (SVR explains 60% of variance in mice; 28–44% in human subgroups where microglial genetics differ). Notably, hFTAA has been shown to attenuate the toxicity of aggregated Aβ species *in vitro*^49^, and was the strongest predictor of neuritic damage in our work. Interestingly, in addition to non-compacted amyloid, we find that the level of plaque-associated microgliosis is a strong predictor of neuritic dystrophy (Figs. 3/4). Considering that the microglial barrier function is believed to protect against neuritic damage, this appears counter-intuitive at first. However, it likely reflects that our quantitative analysis of plaque compaction already captures this beneficial microglial function and separates it from a second, detrimental component of Aβ-driven microglial activation. This aligns with previous studies demonstrating that genetic or pharmacological ablation of microglia in mouse models reduces neuritic dystrophy^45,46^, suggesting that – while microglia generate more inert amyloid through compaction – they are also drivers of plaque-associated neuronal damage through independent inflammatory processes.

Third, our data indicate that clinically used PET-tracers are unable to distinguish neurotoxic from inert amyloid species: We here demonstrate that *ex vivo* amyloid-PET preferentially reports compact plaques and thus misses a sizeable fraction of the non-compacted amyloid that tracks plaque-driven neuronal injury (Fig. 1g). We have previously shown that in mouse models, compacted amyloid shows ~16-fold higher binding of the PET tracer [^18^F]florbetaben compared to diffuse aggregates; accordingly, reducing plaque compaction via *Trem2* knockout measurably reduced [^18^F]florbetaben signal^34^. In contrast, autopsy-validated *in vivo* and comprehensive *ex vivo* PET data using the structurally distinct tracer flutemetamol indicated its binding to less compact plaques and their contribution to the net PET signal^35,36^. However, using *ex vivo* autoradiography, we find that the [^18^F]flutemetamol signal only reflects the decrease in compacted (qFTAA+) amyloid load in 1x/4xLPS-treated mice, missing the increase in non-compacted/filamentous (hFTAA+) amyloid. We found similar results in a small number of samples from sporadic AD patients, in line with a recent autopsy study showing that a PiB-tracer still underestimates plaque burden in patients with high loads of so-called cotton-wool plaques, which lack compact cores and show weak binding of classical amyloid dyes^50^ but are detected by hFTAA^51^. These findings align with the fact that some pathogenic fibrils evade amyloid tracer binding altogether — for example, carriers of the so-called Arctic *APP* mutation (E693G) develop early-onset AD, have heavy Aβ fibril loads yet show little to no PiB retention^52^. Notably, hFTAA can bind aggregates of Arctic Aβ and even alleviate their toxicity *in vitro*^49^.

Thus, together with these previous studies, our data argue that amyloid-PET signals have limited sensitivity to non-compacted amyloid states that drive neuritic dystrophy. Moreover, they indicate that amyloid-PET signals may increase if amyloid becomes more compacted or may look stable while non-compacted amyloid levels and neuritic injury change. Therefore, these studies argue that in clinical trials, amyloid-PET should be complemented by fluid biomarker measures of neuritic injury, such as NfL or pTau181, to ensure full interpretability of amyloid PET readouts.

## Limitations

One limitation of our study is that peripheral LPS injections do not necessarily reflect the complex immunological processes that occur in response to infectious or inflammatory disease, but they were our method of choice, as they provided us with the possibility of inducing temporally defined inflammatory insults. Future studies should include live pathogens, including bacterial and viral species, and determine their impact on plaque morphotype. Another limitation is that autoradiography is *ex vivo* and cannot fully recapitulate *in vivo* PET analysis. Moreover, our human sample sizes are small, and regional sampling may miss heterogeneity. Nonetheless, convergence of our findings across two mouse models, ages, and both sporadica and familial AD tissue, and the consistency of the LCO predictors argue that our main conclusions are robust. Future work should directly compare *in vivo* PET measurements with different tracers to LCO-defined amyloid morphotypes (e.g., via multi-tracer approaches or direct comparison with post-mortem analyses), test microglial-targeted therapeutics with morphotype as a preclinical endpoint, and integrate plaque compaction indices with neuritic injury biomarkers to better forecast clinical decline.

## Methods

### Animals

In this study, hemizygous APP23 transgenic (C57BL/6J-Tg(Thy1-APP_K670N;M671L_)23)^44^ and hemizygous APPPS1 mice (C57BL/6J-Tg(Thy1-APPSw,Thy1-PSEN1*L166P)21Jckr/J)^43^ were used.

APP23 mice express human amyloid-β precursor protein (APP) with the Swedish double mutation under the Thy-1 promoter, and have been backcrossed to C57BL/6J mice for >20 generations. In the neocortex, female mice develop the first amyloid plaques around 6 months of age^44^. Only female mice were used for immune stimulation experiments due to the described significant gender effect on the pathology of cerebral β-amyloidosis^53^, for analyses across the life-span, both male and female mice were analysed.

APPPS1 mice carry two transgenes for APP with the Swedish double mutation and PSEN1 with the PSEN1*L166P mutation, both under the Thy1 promoter. In this model, first plaques start to develop around 6 weeks of age^43^.

All animals were maintained under specific pathogen-free conditions and were housed in groups with enrichment. All experiments were performed in accordance with the veterinary office regulations of Baden-Württemberg (Germany) and were approved by the Ethical Commission for animal experimentation of Tübingen, Germany.

### Peripheral immune stimulation

Female APP23 mice were randomly assigned to treatment groups and were injected at the specified time points (3 months or 7 months) intraperitoneally (i.p.) with LPS (from *salmonella enterica* serotype typhimurium, Sigma) at a daily dose of 500 *μ*g/kg bodyweight. On four consecutive days, animals received either four LPS injections (4xLPS), or four vehicle injections (phosphate buffered saline, PBS), or a single LPS injection followed by three vehicle injections on the following three days (1xLPS).

At the specified time-points (6, 9, or 12 months of age), animals were deeply anaesthetised using sedaxylan/ketamine (64 mg/kg//472 mg/kg). Blood was collected from the right ventricle of the heart, followed by trans-cardial perfusion with ice-cold PBS through the left ventricle. The brain was removed and sagitally separated into the two hemispheres, which were either fixed in 4% paraformaldehyde (PFA) or fresh frozen on dry ice. Fresh frozen hemispheres were homogenised using a Precellys^®^ lysing kit and machine at 20% (w/v) in homogenisation buffer (50 mM Tris pH 8, 150 mM NaCl, 5 mM EDTA) containing phosphatase and protease inhibitors (Pierce). Fixed hemispheres were kept in 4% PFA for 24 h, followed by cryoprotection in 30% sucrose in PBS, and subsequently frozen in 2-methylbutane. For histological analysis, fixed brain hemispheres were coronally sectioned at 25 μm using a freezing-sliding microtome (Leica).

### Patient samples

Formalin-fixed, paraffin-embedded (FFPE) tissue samples were obtained from the frontal cortex of 15 Alzheimer’s disease (AD) patients, including six sporadic AD (sAD) cases and nine familial AD cases carrying mutations in *PSEN1 Intron4* (n=3), *APP V717I* (n=3), *TREM2 R47H* (n=2), and an individual carrying a rare combination of *PSEN1 Intron4* as well as a *TREM2 R47H* mutations (n=1). Detailed patient information is summarized in Supplementary Table 1.

Tissue was obtained from the Queens Square Brain Bank for Neurological Studies, Queen Square Institute of Neurology, University College London, UK, and the work was approved by the institutional ethics review board (Independent Research Ethics Committee of the Medical Faculty, University of Tuebingen; Project Number: 695/2021B02 and the Ethics Committee of the LMU Munich; Project Number: 24–0831_1). Informed consent was obtained from all patients or their families for the use of postmortem tissues for research purposes. Each sample was pseudonymized to protect patient confidentiality.

### Immunostaining

Paraffin-embedded frontal cortex sections from human AD patients were deparaffinized, rehydrated, and subjected to antigen retrieval in citrate buffer (pH 6.0) at 90°C for 35 minutes. Endogenous peroxidase activity was quenched with 0.3% H_2_O_2_ in PBS for 30 minutes. Immunohistochemical stainings were performed using either Vectastain Elite ABC kits (Vector laboratories) or fluorescent secondary antibodies (Jackson Immunolaboratories). For LCO co-staining fluorescent secondary antibodies coupled to Brilliant Violet 421 or Alexa-647 were used for detection of the protein of interest, followed by staining with quadro-formyl thiophene acetic acid (qFTAA) and hepta-formyl thiophene acetic acid (hFTAA) (see below).

Brain sections were blocked for 1 h with 5% normal serum of the secondary antibody species, followed by primary antibody incubation overnight at 4°C. Primary antibodies used were: rabbit anti-Pu.1 (1:1,000; Cell Signaling), rabbit anti-Iba1 (1:1,000; Wako), goat anti-Iba1 (1:500; Novus), rabbit anti-Aβ (NT12; courtesy of P. Paganetti, Basel, Switzerland), rat anti-CD68 (1:1,000; Serotec), sheep anti-Trem2 (1:100; R&D systems), rabbit anti-APP (5313, 1:750; generous gift from C. Haas, Munich) or mouse anti-APP A4 (1:500, Millipore), and rabbit anti-GFAP (1:500, Biozol). Congo Red staining was conducted according to standard procedures. Immunohistochemical images were acquired on an Axioplan 2 microscope; colour images were captured using an Axioplan MRc camera and AxioVision 4.7 software (Carl Zeiss). Fluorescence images were acquired using an LSM 510 META (Axiovert 200M; LSM software 4.2, Carl Zeiss) or Leica TCS SP8 X (LAS X, Leica) confocal microscope with an oil immersion x40/1.3 objective. Sequential excitation of fluorophores ensured no fluorescence cross-talk and best signal throughput. Maximum-intensity projections were generated using IMARIS 8.3.1 software (Bitmap) or Fiji.

### Spectral imaging of LCOs

Free-floating sections were stained with the LCOs qFTAA (1.5 mM in deionized water, diluted 1:500 in PBS) and hFTAA (1.5 mM in deionized water, diluted 1:1000 in PBS for double stain with qFTAA, 1:500 in PBS for single stain) for 30 minutes. After mounting, sections were dried and coverslipped with Fluorsave mounting medium (Calbiochem).

Spectra of qFTAA- and hFTAA-stained amyloid aggregates were acquired on a Zeiss LSM 510 META (Axiovert 200M) confocal microscope equipped with a spectral detector with an oil-immersion x40/1.3 objective. The dyes were excited using the 458 nm argon laser line. Emission spectra were acquired from 470 to 695 nm with steps of 10.7 nm at 3 different regions of interest (ROIs) within the middle core region from an intermediate plane of each plaque. Acquired plaques originated from at least three different sections throughout the mouse brain. The mean emission spectrum per plaque was calculated from the 3 ROIs and normalized to its respective maxima. The ratio of the intensity of emitted light at the blue qFTAA peak (502 nm) and the red hFTAA peak (588 nm) was used as readout for spectral distinction of plaques. For 12 months old APP23 animals, the 502 / 588 nm ratio was calculated for statistical analysis from the normalized mean spectrum generated from at least 35 plaques per animal. Figure 1 shows pooled data from all PBS-injected control animals independent of injection time point. For 9 months old APP23 animals, single staining with hFTAA was conducted and the emission spectrum of 20–30 plaques per animal was acquired and processed as described. The ratio of the intensity of emitted light at the two local emission maxima (545 nm and 588 nm) was used as readout for spectral distinction of plaques.

### Image quantification

For quantification of mean plaque area and Congo red positive area, mosaic images of 5 consecutive sections stained immunohistochemically for NT12 and Congo red were acquired on a Zeiss Axioplan 2 microscope with a x4/0.1 objective. Image analyses were automated using custom-written plugins in Fiji. Quantification of the mean plaque area was performed in the neocortex using the luminance channel. If needed, the gamma value was adjusted to ensure uniform contrast between staining and background. A fixed manual threshold was determined so that plaques were above threshold and was applied to all images. Staining of cerebral amyloid angiopathy and areas with high background staining were excluded from analysis. Only plaques with a minimum size of 30 *μ*m^2^ were included in the analysis. Automatic recognition of plaques was manually checked and corrected upon misclassification. The Congo red positive area was quantified by transforming the RGB image to the CIELAB colour space and subsequently thresholding the positive values of the a* channel, which designates the purity of the red colour.

To quantify neuronal dystrophy, and microglial expression of Iba1, Trem2, and CD68, 5–10 fluorescent images per animal were acquired with the same microscope settings. Detection of qFTAA and hFTAA was achieved using bandpass filters encompassing their respective maximum emission wavelength. Images were subsequently semi-automatically analysed with another custom plugin written in Fiji. Maximum intensity projections were generated to choose the region of interest consisting of the plaque with the desired staining. Fluorescence channels were split and fixed intensity thresholds were applied to each channel. For every plaque, plaque size and area of the costained protein within the region of interest were determined based on thresholded areas. Plaques smaller 100 *μ*m^2^ or bigger 2500 *μ*m^2^ were excluded from analysis. The area of the protein of interest was divided by the plaque size for normalization purposes. To analyse colocalization between the protein of interest and the amyloid plaque, the same images were analysed in IMARIS. Using the 3D colocalization tool, a constant threshold was applied to the fluorescent channels for hFTAA and the protein of interest. The threshold for hFTAA was determined so that the whole amyloid plaque (i.e. including the qFTAA positive core) was included in the colocalization analysis. The percentage of colocalized plaque material in relation to total plaque material per image was used as readout for protein colocalization. To assess the influence of plaque size and structure on neuronal dystrophy, acquired images from PBS-treated 12 months old APP23 controls were semi-automatically analyzed with a custom plugin written in Fiji. Maximum intensity projections were generated to choose the region of interest consisting of the plaque with the surrounding APP staining. For every plaque, a core region was chosen, wherein the intensity of qFTAA and hFTAA was subsequently measured. Fluorescence channels were split and fixed intensity thresholds were applied to each channel. Based on thresholded areas, plaque size and the ratio of qFTAA intensity to hFTAA intensity in its core were determined for every plaque (core qFTAA / hFTAA ratio), in addition to the area of surrounding APP staining.

### Support vector regression

We developed a model for APP area prediction using support vector regression (SVR). Separate models were constructed for human and mouse datasets. For each model, APP area served as the response variable, and sex, age, plaque-associated Iba1 area, qFTAA, hFTAA, and the Core qF/hF ratio were included as predictors. For mouse data analysis, samples were randomly partitioned at the animal level into training and testing sets, and five-fold cross-validation was performed for model evaluation. For the human data analysis, the SVR model was trained exclusively on data from sporadic AD, *PSEN1 Intron4* and *APP V717* carriers, with this data set further split into training and testing sets for model fitting and validation. The trained model was subsequently applied to the *TREM2* mutation carriers to assess cross-group generalizability. Model performance was quantified using the coefficient of determination (R^2^) between observed and predicted APP area in the testing set.

### Stereological quantification

Stereological quantification was performed by a blinded observer on random sets of every 12^th^ systematically sampled 25 μm thick sections throughout the neocortex. Analysis was conducted with the Stereologer software (Stereo Investigator 6; MBF Bioscience) and a motorized x-y-z stage coupled to a video microscopy system (Optronics). For quantification of microglial numbers based on Pu.1 staining, the optical fractionator technique was used with three-dimensional disectors as previously described^54^. The number of plaque-associated GFAP- and Pu.1-positive cells was determined for at least 30 plaques per animal. Plaque load was determined using the area fraction fractionator technique^55^ based on Congo Red and anti-Aβ staining (NT12 antibody).

### ELISA

For quantification of Aβ by ELISA, brain homogenates were pretreated with formic acid (Sigma-Aldrich, final concentration: 70% vol/vol), followed by sonication for 30 seconds on ice, and subsequent centrifugation at 25,000 g for 1 hour at 4°C. Supernatants were equilibrated in neutralization buffer (1 M Tris base, 0.5 M Na_2_HPO_4_, 0.05% NaN_3_ (wt/vol)). Aβ was measured using human (6E10) Aβ triplex assay (Meso Scale Discovery, MSD) according to the manufacturer’s instructions. Total Aβ was calculated as the sum of the measured values for Aβ_1–38_, Aβ_1–40_, and Aβ_1–42_.

For cytokine measurements, brain homogenates were centrifuged at 25,000 g for 30 minutes at 4 °C. Supernatants were analysed with mouse pro-inflammatory panel 1 V-plex plate (MSD) according to the manufacturer’s instructions. To determine blood cytokines, serum was obtained by coagulation of whole blood in Vacuettes (Greiner Bio-One) for 10 min at room temperature, followed by centrifugation for 10 min at 2,000 g. Serum samples were diluted 1:2 before cytokine measurement. The investigator was blinded to the treatment groups.

Measurements were performed on a Mesoscale Sector Imager 6000 and data were analyzed using MSD discovery workbench software 2.0. For brain homogenates, cytokine levels were normalised against total protein amount as measured by microplate Pierce bicinchoninic acid (BCA) protein assay (Perbio Science).

### Western Blotting analysis

For Western Blotting, Urea was added at a final concentration of 5.4 M to total brain homogenates. Samples were incubated for 10 minutes at 70°C, followed by centrifugation for 1 minute at 16000 g. Pellets were discarded and protein levels of the supernant were quantified with a microplate Pierce 660 nm protein assay (Thermo Fischer) and adjusted to equal protein concentrations. After addition of Urea sample buffer (final concentration: 10% glycerol (vol/vol), 2% SDS (wt/vol), 0.0002% Bromphonel blue, 0.1M Tris-HCL (pH 8.6), 2% β-mercaptoethanol (vol/vol)) 15 μg total protein per sample were analysed on BOLT 4–12% Bis-Tris gels (Thermo Fischer) using standard procedures. Proteins were transferred to nitrocellulose membranes, followed by boiling of the membranes for 5 minutes in a microwave. Ponceau S staining was conducted to verify equal protein transfer across samples. Blocking was performed with 5% milk in phosphate buffered saline containing 0.05% Tween (PBST) for 1h and blots were incubated with the following primary antibodies: mouse anti-Aβ (6E10; 1:2500, Covance Research Products), mouse anti-GAPDH (1:10^6^, Acros Antibodies) in PBST overnight at 4°C. Membranes were then probed with the respective secondary HRP-labelled antibodies (1:20,000, Jackson ImmunoLaboratories). Protein bands were detected using chemiluminescent peroxidase substrate (ECL prime, GE Healthcare). Densitometric values of protein band intensities were determined in Fiji and normalised to GAPDH intensities. Samples were analysed at least three times on separate blots. The mean value of normalised intensities of all technical replicates per sample is shown and was used for statistical analysis.

### Amyloid-PET Autoradiography

In vitro autoradiography on human and murine brain sections was performed with a solution of 0.4 MBq/mL [^18^F]Flutemetamol in PBS. The sections were fully covered with tracer solution and incubated for one hour. After incubation, the sections were washed with PBS, 70/30 EtOH/PBS and 30/70 EtOH/PBS and then dried at room temperature for one hour. The brain sections were exposed to a phosphor imaging plate (BAS-IP MS 2025 E, GE) for at least 10h in the dark, which was then scanned with a CR-Reader (CR35 BIO, DÜRR MEDICAL). Evaluation of the images was performed using Aida Image Analyzer software (v.4.50.010, Elysia-raytest GmbH). A manually drawn region of interest (ROI) was placed in the white matter for reference. After background subtraction, intensity normalization was performed by calculating cortex to white matter ratios.

### Statistical analysis

Linear regressions were performed using JMP software (version 14.2.0 or higher). If necessary, data were first log10 or log10[x+1]-transformed to achieve a normal distribution. Data were then analysed using the ‘Fit model’ function, generating parameter estimates as well as residual vs. leverage plots, where a least squares line (red) and confidence bands (shaded red) provide a visual representation of the statistical significance (at the 5% level) of the effect of X; a significant effect is evident by the crossing of the confidence lines (shaded red/red) through the blue line in the graph, which indicates the mean of the Y leverage residuals. To calculate the data points in the graph, the mean value of Y is added to the Y-residuals and the mean of the X-value is added to the X-residuals, generating “leverage residuals”, and these pairs of residuals are then used to generate the effect leverage plots shown^56^.

All other statistical analyses were performed using Prism 10. Data were assessed for normal distribution (Shapiro-Wilk test) and statistical outliers (ROUT method, Q = 0.5%). The Brown-Forsythe test was used to check equality of variances. If the normality criterion was met and variances were not significantly different, data were analysed using a one-way ANOVA, followed by pairwise comparison (if P<0.05) with post-hoc Tukey correction. For data sets with small sample size or non-normally distributed data, the Kruskal-Wallis test was performed, followed by pairwise comparisons (if P< 0.05) with post-hoc Dunn’s correction. For comparisons between two groups, two-tailed Mann Whitney test was performed. All experiments were at least performed twice or in independent batches of animals (figures show the pooled data).

## Extended Data

## Supplementary Material

Supplementary Files

This is a list of supplementary files associated with this preprint. Click to download.

- Supplementarytable1patientsamplesinfo.xlsx

## Significance statement

Systemic inflammatory insults durably reprogram microglia and remodel Aβ plaques. In APP23 mice, LPS treatment weakens microglial plaque engagement, leading to more non-compacted amyloid and increased neuritic dystrophy. Across aging cohorts, a second mouse model, and human AD tissue, non-compacted amyloid and plaque-associated microgliosis consistently predicted neuritic damage, whereas compacted amyloid correlated weakly or inversely. *Ex vivo* amyloid-PET tracked compact plaques but not non-compacted amyloid, revealing a diagnostic gap. Our results unify genetic (*TREM2/APOE*) and environmental risks via impaired microglial-plaque interaction and argue for biomarkers and therapies targeting non-compacted amyloid states and microglial plaque containment.
